# NONATObase: a database for Polychaeta (Annelida) from the Southwestern Atlantic Ocean

**DOI:** 10.1093/database/bau002

**Published:** 2014-02-24

**Authors:** Paulo R. Pagliosa, João G. Doria, Dairana Misturini, Mariana B. P. Otegui, Mariana S. Oortman, Wilson A. Weis, Larisse Faroni-Perez, Alexandre P. Alves, Maurício G. Camargo, A. Cecília Z. Amaral, Antonio C. Marques, Paulo C. Lana

**Affiliations:** ^1^Departamento de Geociências, CFH, Universidade Federal de Santa Catarina, 88040-970 Florianópolis, Santa Catarina, Brazil, ^2^Núcleo de Estudos do Mar, CCB, Universidade Federal de Santa Catarina, 88040-900 Florianópolis, Santa Catarina, Brazil, ^3^Que?Art, 80.520-590 Curitiba, Paraná, Brazil, ^4^Centro de Estudos do Mar, Universidade Federal do Paraná, 83255-000 Pontal do Sul, Paraná, Brazil, ^5^Departamento de Zoologia, Instituto de Biologia, Universidade Estadual de Campinas, 13083-970 Campinas, São Paulo, Brazil and ^6^Instituto de Biociências, Universidade de São Paulo, 05508-090 São Paulo, Brazil

## Abstract

Networks can greatly advance data sharing attitudes by providing organized and useful data sets on marine biodiversity in a friendly and shared scientific environment. NONATObase, the interactive database on polychaetes presented herein, will provide new macroecological and taxonomic insights of the Southwestern Atlantic region. The database was developed by the NONATO network, a team of South American researchers, who integrated available information on polychaetes from between 5°N and 80°S in the Atlantic Ocean and near the Antarctic. The guiding principle of the database is to keep free and open access to data based on partnerships. Its architecture consists of a relational database integrated in the MySQL and PHP framework. Its web application allows access to the data from three different directions: species (qualitative data), abundance (quantitative data) and data set (reference data). The database has built-in functionality, such as the filter of data on user-defined taxonomic levels, characteristics of site, sample, sampler, and mesh size used. Considering that there are still many taxonomic issues related to poorly known regional fauna, a scientific committee was created to work out consistent solutions to current misidentifications and equivocal taxonomy status of some species. Expertise from this committee will be incorporated by NONATObase continually. The use of quantitative data was possible by standardization of a sample unit. All data, maps of distribution and references from a data set or a specified query can be visualized and exported to a commonly used data format in statistical analysis or reference manager software. The NONATO network has initialized with NONATObase, a valuable resource for marine ecologists and taxonomists. The database is expected to grow in functionality as it comes in useful, particularly regarding the challenges of dealing with molecular genetic data and tools to assess the effects of global environment change.

**Database URL**: http://nonatobase.ufsc.br/

## Introduction

Internet resources integrating different data related to biodiversity are friendly and widespread nowadays. Several examples of regional and global databases for marine biodiversity are available, such as the Ocean Biogeographical Information System (IOC; http://www.iobis.org), SeaLifeBase (www.sealifebase.org; all global taxa), Atlas of Living Australia (http://www.ala.org.au; Australian Polychaeta), MacroBen (1) (European benthic taxa), Manuela database (2) (global meiobenthos and nematodes), FishBase (www.fishbase.org; global fishes), Hexacoralia (http://www.kgs.ku.edu/Hexacoral; global sea-anemones) and AlgaeBase (http://www.algaebase.org; global algal taxa). These geographic database resources allow assessment of large-scale patterns of biodiversity. However, the construction of organized and useful data sets designed for a diverse array of users faces many challenges, from data acquisition and standardization to accurate and efficient outputs.

The fundamental first step for designing and building a biodiversity data set is to survey and compile past and present biodiversity information. Many early studies and grey literature are difficult to retrieve, whereas current data are more readily accessible in scientific e-journals and abstract indexes and data repositories of research funding agencies and governments. Furthermore, personal relationships are a main social component in research networks, and compiling a database is made much easier if the contributing scientists are willing to share their data (1, 3).

The willingness to contribute was the basis of the NONATO network, an association of Brazilian, Uruguayan and Argentine scientists sharing a common interest—research on Polychaeta (Annelida). In the past 15 years, expertise and knowledge of polychaetes from Southern South America has become greater than that found for other marine benthic invertebrates (4–6). Despite the growing research rates, reflected, for instance, in the larger number of published papers on polychaetes, collaborative investigations at continental scales are still scarce. Trying to attenuate this problem, we have built a database focusing on the polychaetes of the SouthWestern Atlantic Ocean (SWAO)—the integrated NONATObase. This database is named after Prof. Edmundo Ferraz Nonato, a pioneer in oceanographic studies in the South Atlantic that mentored several generations of marine biologists, mainly polychaetologists like himself. The aim of the NONATObase is to compile, organize and share all available taxonomy and ecology data related to Polychaeta of the SWAO (also encompassing areas near the Antarctic), from 5°N (Brazil’s Northern limit) to 80°S (Antarctic Peninsula and Weddell Sea). Our specific goals are (i) to put all the information in a unique data set allowing synergy among information of different natures; (ii) to make the information available in an on-line and user-friendly environment; (iii) to operationalize the use of data through spreadsheets and maps of species distribution and abundances; and (iv) to enhance collaborative studies developing friendly tools for sharing information.

The geographic and temporal ranges of this initiative require reliable and well-organized means to manage large amounts of data. One of the main difficulties in organizing this information is the diversity of the primary data, which comes from different scientific areas, sites and research teams with their own methods for organizing data (7). The development of a database, in this case, is important for integrating and ensuring data quality under common taxonomic, geographical and sampling standards, but highly dependent on the collaboration of the researchers and institutions hosting the primary information (1, 2, 8–10). Evidently, the advantage is to allow researchers to test hypotheses using a collection of data otherwise unavailable if based on individual efforts (11), enhancing explanatory and consistency powers of the taxonomic and ecological inferences.

Therefore, our goal is to describe the content, structure, data handling, tools, functionality and data policy of the NONATObase.

### Governance of the database

The NONATObase is hosted on a web server maintained by Universidade Federal de Santa Catarina (UFSC) and under the curation of Paulo R. Pagliosa. UFSC is responsible for support in infrastructure and technical staff. The curator is formerly responsible for data gathering, data digitization, data wrapping in metadata and making it available for sharing. Data control was carried out by a group of designated regulators to ensure correct spelling of scientific names, reliable coordinate locality and data classification used. Taxonomic validation of regional species names and the establishment of criteria to qualify the reliability of species identification were made by a taxonomy committee formed by members of the NONATO network. NONATObase is under Creative Commons—Attribution-NonCommercial-NoDerivs 3.0 Unported License.

### Content and structure of the database

**Figure 1.**
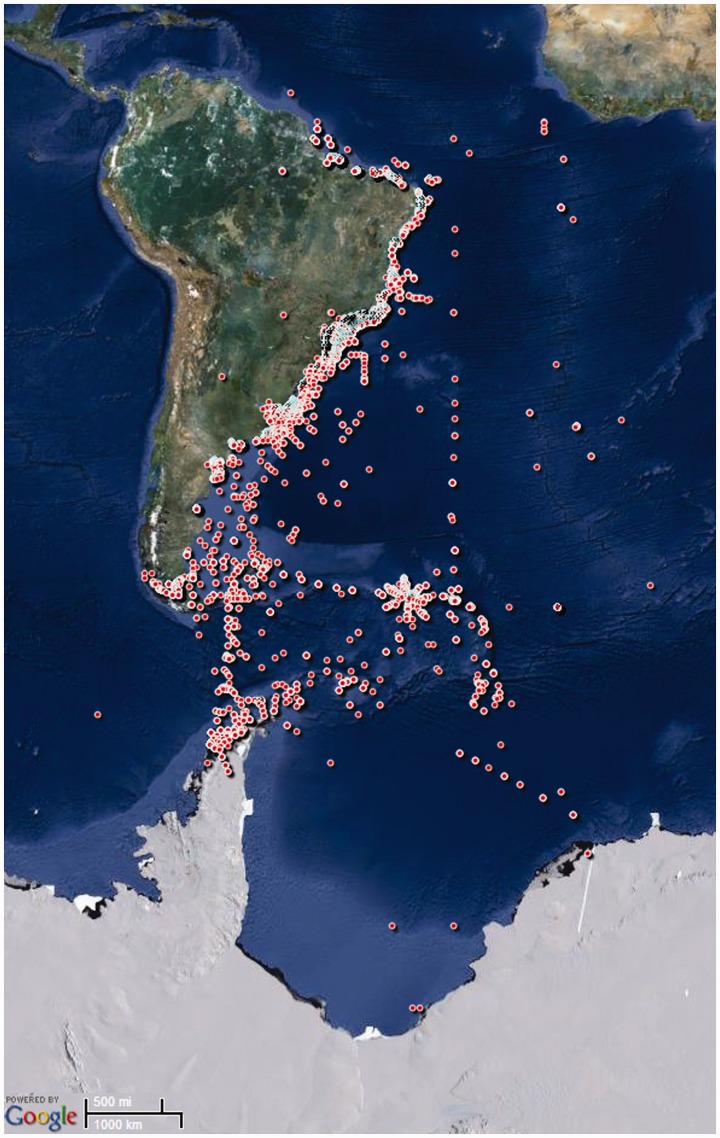
Geographical data of the sampling stations included in the NONATObase.

Primary information used to feed the database was based on several sources. Exploratory surveys using ‘polychaet*’ or ‘marine benth*’ and ‘Brazil’ or ‘Argentina’ or ‘Uruguay’ or ‘Chile’ or ‘Antarctic’ were carried out in the Web of Knowledge (http://www.webofknowledge.com), Scopus (http://www.scopus.com) and Scielo (http://www.scielo.org). Concomitantly, scientific theses were surveyed in the CAPES databank of Brazilian theses (http://capesdw.capes.gov.br/), accessing information of all graduate programs in zoology, aquatic science, oceanography and ecology. Data were also surveyed from the Brazilian on-line curriculum databank (http://lattes.cnpq.br/) for national polychaete researchers and their successive generations of students. Additionally, collaborators from 28 research institutions surveyed their libraries and institutions for non-indexed literature, such as papers, theses, dissertations, monographs, unpublished reports and museum data. All documents were filtered based on two types of accepted data: (i) species name and locality or (ii) abundance data on taxonomic resolution of species, genus or family and locality. This procedure resulted in 1150 references from 1859 to early 2013, geographically ranging from 4.5°N to 77°S and from 82°W to 1.4°W (Figure 1), which were used to feed the NONATObase. New data to be incorporated in the database will be provided by the NONATO technical network with an annual survey for references.

**Figure 2.**
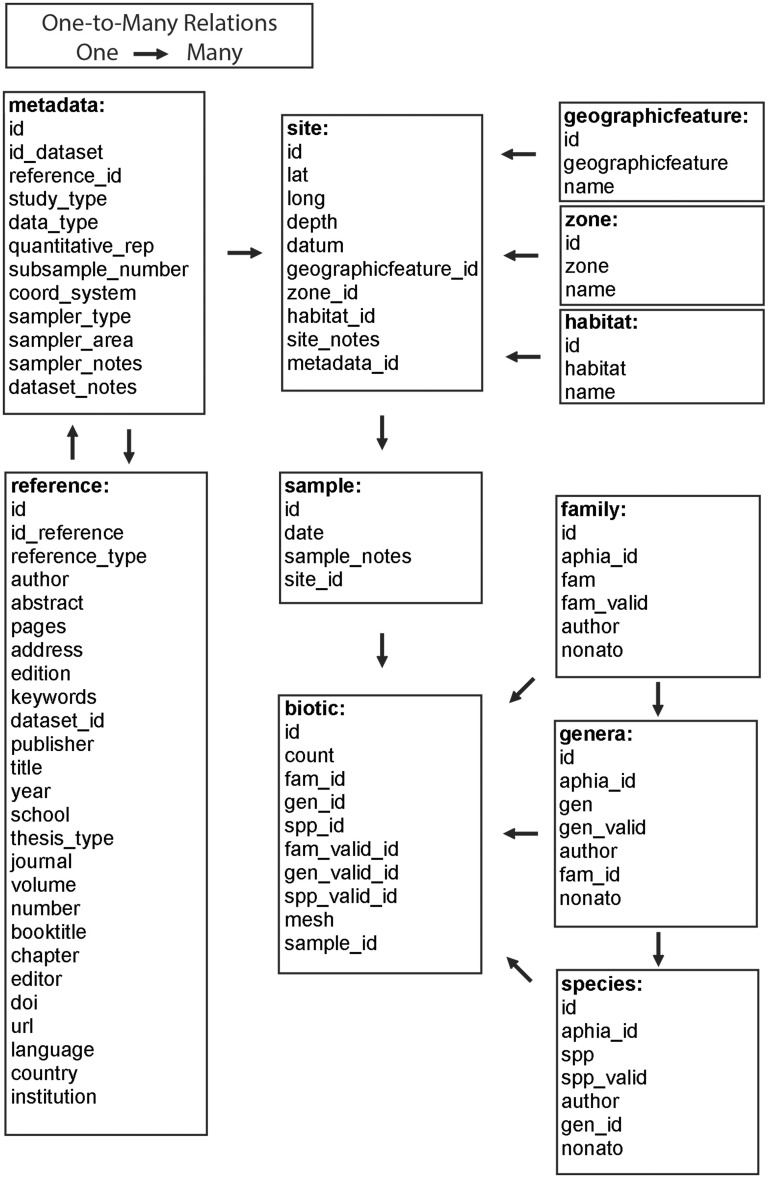
Relational structure of the integrated NONATObase.

NONATObase was built as a relational object-oriented database developed using Structured Query Language (MySQL) and Hypertext Preprocessor (PHP), and hosted on a Server running a GNU/Linux operational system maintained by Universidade Federal de Santa Catarina (http:/nonatobase.ufsc.br). The database design seeks maximize interrelation of the data, allowing filtering by almost all parameters within it. The design was conceived in 11 tables (Figure 2) in which field names were designated in a self-explanatory manner. Table ‘metadata’ identifies information regarding study, references, sites and samples that originated the data set. Each field of that table was related to the many fields of the table ‘reference’ and ‘site’. Table ‘reference’ shows fields generally used to manage references. Table ‘site’ contains information on latitude, longitude, datum and depth. It is linked to the classification used in the tables ‘geographical feature’, ‘zone’ and ‘habitat’, and is also related to the table ‘sample’. Table ‘sample’ individualizes the sampling in each site, adding information on the date of sampling, and is related with the many fields of table ‘biotic’. The abundance and occurrence data for each taxa sampled are determined by a registry in table ‘biotic’. This table contains information on individual count, mesh size used and the relational fields for the taxa name and the sample it pertains to. Each field of this table is linked with the classification used in the tables ‘family’, ‘genus’ and ‘species’. These tables identify the taxa in each taxonomic level, and all have information on the name of the taxa recorded in the reference, valid name of the taxa, authorship of the taxa, AphiaID and NonatoID.

## Data Handling

Once all organismic biological information is indexed by a scientific species name, the quality of the taxonomy used is of ultimate importance for any biological data set. NONATObase adopts a unique identity (NonatoID) for each species name that is associated to the AphiaID of World Registry of Marine Species/World Polychaete Database - WoRMS (http://www.marinespecies.org/polychaeta). Therefore, a taxonomy committee was created to solve the persistent taxonomic issues related to the misidentification and status of some species. The taxonomy committee of the NONATObase verifies users’ demands on possible misidentifications and nomenclatural uncertainties based on established taxonomy grounds, and helps to establish identification confidence levels. Additionally, members of this committee develop taxonomic research to clarify the taxonomic status of several polychaete species from the southwestern Atlantic that shall be incorporated by the NONATObase.

Database feeding procedure avoided data redundancy by identifying each primary reference by a unique ID (id_reference) related to its own data set (id_dataset). References were then listed for each data set they were related to. An information set was extracted from each reference, transformed into individual standardized spreadsheets that were triple checked and finally automatically uploaded to the NONATObase. Species data were recorded preserving the original scientific names cited in the reference, and updated whenever necessary. The database provides authorship of the species name and synonymy. Whenever possible, data on the relative abundance for benthic species are standardized as a mean value of a sample unit per square meter (m^2^) obtained from a given number of subsamples. Abundance data are also related to different taxonomic levels, allowing searches based on family, genera and species levels among and within data sets.

Collecting dates of the biological samples were recorded; whenever this information was not available, we included the year of the reference as proxy and indicated this in the observation. The georeference for each species or samples was indicated in decimal degrees using the geodetic WGS84 datum. Original data were classified wherever possible according to the type of study (taxonomy or bioecology), data sampling (qualitative or quantitative), geographical feature, zone and habitat (Table 1), sample depth (m), subsample number, sampler area (m^2^), sampler device (core, quadrat, trawl, grab, dredge, box-corer, by hand or other) and mesh size (mm) used to sort the polychaetes.

**Table 1.** Classificatory framework used by NONATObase

Geographical feature	Zone	Habitat
Continental shelf (0 m up to 129 m deep)	Intertidal	Artificial
		Consolidated
		Mangrove
		Parasitic or symbiotic
		Prey
		Saltmarsh
		Soft bottom
	Sublittoral	Artificial
		Consolidated
		Parasitic or symbiotic
		Pelagic
		Prey
		Soft bottom
		Vegetated
Continental slope (>130 m deep)	None	Artificial
		Parasitic or symbiotic
		Pelagic
		Prey
		Soft bottom
Estuary	Intertidal	Artificial
		Consolidated
		Mangrove
		Parasitic or symbiotic
		Prey
		Saltmarsh
		Soft bottom
	Sublittoral	Artificial
		Consolidated
		Parasitic or symbiotic
		Pelagic
		Prey
		Soft bottom
		Vegetated
Coastal lagoon	Intertidal	Artificial
		Consolidated
		Mangrove
		Parasitic or symbiotic
		Prey
		Saltmarsh
		Soft bottom
	Sublittoral	Artificial
		Consolidated
		Parasitic or symbiotic
		Pelagic
		Prey
		Soft bottom
		Vegetated
Freshwater	None	River
		Artificial
		Parasitic or symbiotic
		Prey

### Database tools and functionality

**Figure 3.**
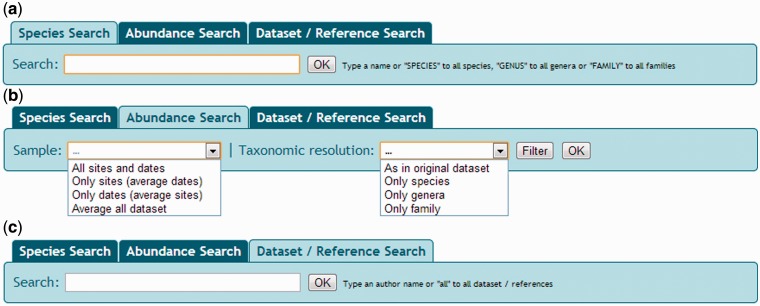
Snapshot of NONATObase for species (a), abundance (b) and data set/reference (c) queries.

**Figure 4.**
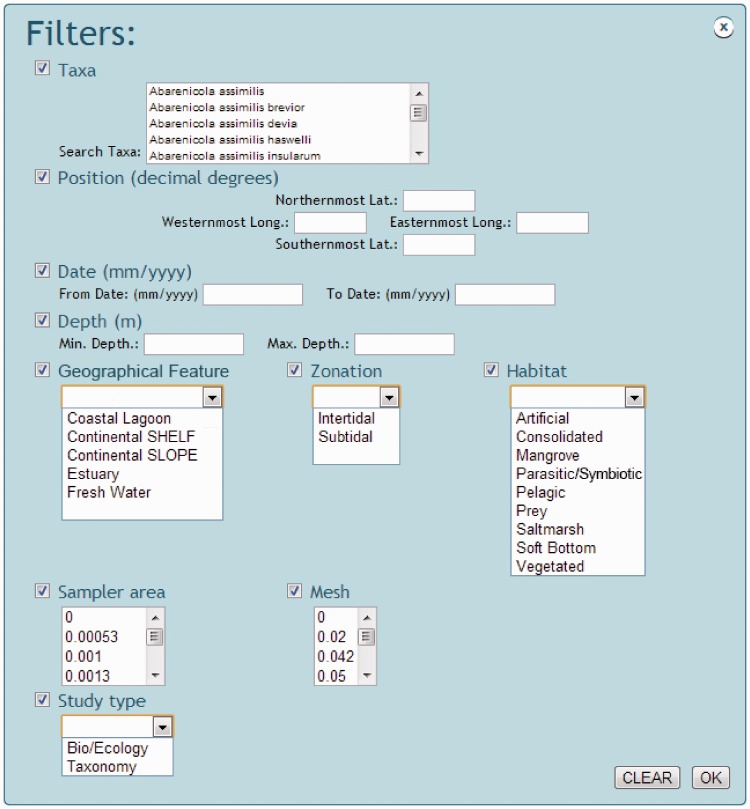
Snapshot of NONATObase filters in query for species and abundance.

The NONATObase web application allows access to the data from three different perspectives: species, abundance and data set/reference (Figure 3). Species queries can be performed based on specific taxa, all species, all genera or all families. Broader searches (i.e. all species) of a given group of taxa are also available. Geographical ranges may be defined producing distribution maps of the selected data (i.e*.* taxa or data sets). Additionally, users may access the taxon name register of WoRMS. Filters are accessible for specifying ranges of geographical position, date and depth, or using a menu list of types of geographical feature, zone, habitat and type of study (Figure 4). All selected data, data sets, maps and references based on the query performed maybe both visualized and exported.

Abundance data may be accessed using three basic alternatives (Figure 3). First, querying for taxonomic resolution as in the original data set (maybe with variable resolution), only species, only genera or only family level. Second, searching for samples pathway of all sites and dates, only sites, only dates or average data set (between all samples and dates). Third, searches can be filtered from a list of taxa within the taxonomic resolution first specified, the preferred range of geographic position, date and depth, and preferred geographical feature, zone, habitat, sampler area (i.e*.* the amount of area that has been sampled by a given tool, like a trawl or a core) and mesh size (Figure 4).

Queries are accessible for a specific data set or for all data sets of the database (Figure 3). Metadata summaries, including details of the references, type of study, data collector, subsample numbers, sampled area, geographical feature, zone, habitat, mesh size, dates of the study and range of depth and position, are accessible before browsing data set domains. References are listed according to the data set they belong to.

**Figure 5.**
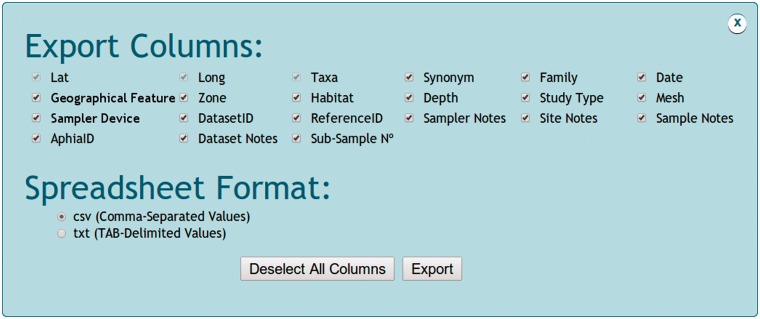
Snapshot of NONATObase exportation options.

All data, maps and references from the specified species, abundance or data set query can be visualized and exported. Data export can generate different types of data frames that vary according to the selected data search (Figure 5). Species searches generate an x (longitude), y (latitude) and z (taxa) spreadsheet format. Abundance searches generate a variable (taxa) *vs* sample (longitude/latitude) spreadsheets format. Despite the query mode, users can choose the columns to be included in the output spreadsheet. Columns bring information on longitude, latitude, taxa, synonymy, family, date, geographical feature, zone, habitat, depth, study type, mesh, sampler device, sampler area, subsample number, data setID, referenceID, AphiaID, NonatoID, sampler notes, site notes and sample notes. Files can be exported as ‘.csv’ (comma-separated values) or ‘.txt’ (tab-delimited values) format. Maps of taxa distribution accessed via species, abundance or data set queries can be exported as ‘.pdf’ files. Each or all references selected in the different queries performed can be exported as a ‘.bib’ file.

## Data Policy

Data of the NONATObase are entirely compiled based on scientific references, and they are not a property of the NONATO network. The guiding principle of the base is to maintain free and open access to data, always based on stimulated or induced scientific partnerships. Any researcher can share data with the NONATObase, and collaborators can freely access and download data. Visitors can freely access and consult the website but need consent to download data. By downloading data, users are requested to confirm that they have read and agreed with the user statement before they are granted access to the data: (i) if data are extracted from the database website for secondary analysis resulting in a publication, the NONATObase should be cited; (ii) if any individual data source of the NONATObase is essential to reach a given conclusion or if data constitute >10% of the records used in a secondary analysis, the individual data source should also be cited and (iii) if the downloaded data (map or spreadsheet) from the NONATObase website result in a publication, it should be sent to the NONATO network as a citation or file.

### Concluding remarks

The NONATObase was designed to provide conditions for sharing and exchanging data, allowing research communication, data mining, reuse and review, which are essential to enhance scientific productivity, collaboration and discoveries. The NONATO network is a valuable e-science resource for marine ecologists and taxonomists from South America and worldwide. Sustainable use and management of biodiversity requires that biological information is quickly and freely made available to decision makers, stakeholders and scientists (12). In this sense, databases cannot supersede non-existent data, but they can point when and mainly where attempts to get information should be driven.

Data archaeology and recovery enhance species distribution and taxonomy knowledge using low cost methods (13). To achieve this, authors need to be careful when providing information in research articles, on the integrity of the data and on the transparency of the data acquisition (i.e*.* sampling date, sample size and geographical position), ensuring the quality of the data recovered. Presently, constraints related to space for complete lists of taxa and abundance tables within publications are surpassed or greatly reduced with the possibilities provided by the e-journals.

NONATObase mostly differs from other biodiversity database, including other polychaete database (i.e*.* Atlas of Living Australia), by including abundance data. Only data sets based on expeditions and master projects (i.e. Macroben) made available quantitative data. Otherwise, species distribution in NONATObase is constructed similarly to other databases, and thus their data can be merged to analyze broader geographical scales. Global initiatives, such as the Ocean Biogeographical Information System (OBIS), will benefit from NONATObase because the geographical area covered, the Southwestern Atlantic, is among the poorest represented for polychaete data. The NONATObase is considered to be the first step of a more extensive process. The database is expected to interact with users, making it more functional as time passes. Future steps will include the incorporation of sets of environmental data-like sediment texture, eutrophication (dissolved nutrients) and pollutant (heavy metals, hydrocarbons) variables to permit pairwise analysis. We will also integrate interactive keys and molecular genetic data for the polychaete species of the Southwestern Atlantic to the data set, besides incorporating tools to assess the effects of global climate change.
